# The Interpretation of Scholars' Interpretations of Confidence Intervals: Criticism, Replication, and Extension of Hoekstra et al. (2014)

**DOI:** 10.3389/fpsyg.2016.01042

**Published:** 2016-07-08

**Authors:** Miguel A. García-Pérez, Rocío Alcalá-Quintana

**Affiliations:** Departamento de Metodología, Facultad de Psicología, Universidad Complutense, Campus de SomosaguasMadrid, Spain

**Keywords:** method bias, confidence intervals, hypothesis testing, parameter estimation, statistical education

## Abstract

Hoekstra et al. (*Psychonomic Bulletin* & *Review*, 2014, *21*:1157–1164) surveyed the interpretation of confidence intervals (CIs) by first-year students, master students, and researchers with six items expressing misinterpretations of CIs. They asked respondents to answer all items, computed the number of items endorsed, and concluded that misinterpretation of CIs is robust across groups. Their design may have produced this outcome artifactually for reasons that we describe. This paper discusses first the two interpretations of CIs and, hence, why misinterpretation cannot be inferred from endorsement of some of the items. Next, a re-analysis of Hoekstra et al.'s data reveals some puzzling differences between first-year and master students that demand further investigation. For that purpose, we designed a replication study with an extended questionnaire including two additional items that express correct interpretations of CIs (to compare endorsement of correct vs. nominally incorrect interpretations) and we asked master students to indicate which items they would have omitted had they had the option (to distinguish deliberate from uninformed endorsement caused by the forced-response format). Results showed that incognizant first-year students endorsed correct and nominally incorrect items identically, revealing that the two item types are not differentially attractive superficially; in contrast, master students were distinctively more prone to endorsing correct items when their uninformed responses were removed, although they admitted to nescience more often that might have been expected. Implications for teaching practices are discussed.

## Introduction

In a recent study, Hoekstra et al. (2014) administered a questionnaire to first-year students, master students, and researchers, asking them to indicate whether or not each of six interpretations (all of them incorrect) follows logically from Prof. Bumbledorf's claim that “the 95% CI for the mean ranges from 0.1 to 0.4.” Respondents were asked to answer all items and Hoekstra et al. reported similarly high endorsement rates in all groups, from which they concluded that misinterpretation of CIs is robust. Yet, method bias may have produced this outcome artifactually, as discussed next.

First, by including only items that express incorrect statements about a CI, endorsement always points in the direction of presumed misinterpretation, with correct interpretation inferred indirectly only if none of the items is endorsed. A balanced number of correct and incorrect items is needed to distinguish true misinterpretation (when incorrect items are endorsed and correct items are not) from confusion or incognizance (when incorrect and correct items are endorsed equally often).

Second, when all items are incorrect, the request to answer all items aggravates the problem: Respondents who ignore what CIs are (e.g., first-year students who have not taken an inferential statistics course) or who are uncertain as to how it is interpreted (arguably, some master students and researchers) are forced to provide responses that will be construed as misinterpretation, except in the unlikely event that such respondents opt to endorse none of the items (an eventuality that, for the same reason, cannot be construed as reflecting correct interpretation of a CI). Providing a third response option (i.e., “I don't know”) and allowing omissions is needed to separate true misinterpretations from mere guesses forced by the request to answer all items.

Third, how scholars interpret CIs refers to their natural verbalizations, which are not adequately represented by the true/false items in a questionnaire. For instance, first-year students will surely make no spontaneous attempt at interpreting something that they have never heard of (e.g., a CI reported in a paper on their reading list for some course); similarly, active researchers' interpretation of a CI can only be truly assessed via analysis of their spontaneous descriptions of the meaning of the CI they are reporting, or maybe also by analysis of their open-ended responses when asked to describe the interpretation of a CI. Admittedly, data for such assessments are much harder to obtain and even harder to interpret, but inferences are certainly suspect when data come from scholars' responses to items whose wording does not match the wording that they would have used in the statement they might have produced.

Finally, it is contentious that all items were incorrect, as demonstrated by the exchange between Miller and Ulrich (2016) and Morey et al. (2016). For another example, item 3 stated that “the null hypothesis that the true mean equals 0 is likely to be incorrect” (see the Appendix in Supplementary Material). This statement cannot be tagged as incorrect without clarification of what “likely” means in it, something that is left to each respondent's discretion. Also, that the true mean has some precise value is surely (let alone likely to be) incorrect, which makes the statement on the item correct and, thus, endorsable by a knowledgeable respondent. It is also arguable that all items assess interpretation of CIs. For instance, item 6 stated that “if we were to repeat the experiment over and over, then 95% of the time the true mean falls between 0.1 and 0.4.” By referring to a true mean that varies across repetitions of the experiment, the item is immediately classifiable as incorrect by anyone who understands that the population mean is a fixed and invariant value; endorsing this item is thus indicative of a more basic misconception that does not involve CIs.

To address these issues, we replicated Hoekstra et al.'s (2014) study using a questionnaire that included the same items and two more expressing correct interpretations of CIs (see the Appendix in Supplementary Material). We limited our study to first-year and master students, in search for traces of knowledgeability in statistically educated individuals in comparison to still uneducated first-year students. As in the original study, respondents were asked to answer all items. Yet, on an immediate second pass that had not previously been mentioned, master students were asked to mark the items that they would rather have omitted or to which they would have responded “I don't know” if they had had the option. This second pass was omitted with first-year students because they did not know what a CI is or how it is computed. Our results reflect first-year students' incognizance in that they endorsed correct and incorrect items identically, revealing also that the two item types do not differ in attractiveness and, thus, ruling out a potential source of method bias. Master students showed instead distinct endorsement patterns for incorrect and correct statements, with higher proneness to endorse the latter. Self-declared incognizance or preference for omitting on the second pass was not uncommon among master students. Restricting the analysis to informed responses only, the patterns of endorsement of correct and incorrect statements by master students separated even further.

The paper is organized as follows. The next section discusses the two correct interpretations of CIs, leading to an analysis of the (un)certainty with which misinterpretation of CIs can be inferred from endorsement of the items in Hoekstra et al.'s (2014) questionnaire. Next, we analyze and discuss a conspicuous difference between first-year and master students in the data of Hoekstra et al., which is consistent with the notion that master students may have used knowledge of CIs to disambiguate nominally incorrect items and identify them as correct statements. Finally, we describe our extension and discuss the results.

## Two correct interpretations of a CI

The theory from which CIs are derived and the ensuing properties of CIs permit two interpretations that we will separately describe and discuss next. One is in the context of significance testing; the other is in the context of parameter estimation. With such correct interpretations in mind, we will close this section with a discussion of the resultant difficulty to infer misinterpretation of CIs from endorsement of the items in the questionnaire of Hoekstra et al. (2014).

### The CI as the range of point hypotheses that the current data will not reject in a size-α test

The expressions with which the upper and lower limits of the CI for a distributional parameter are computed emanate from the setup of a significance test for that parameter. The link holds for any parameter but, for simplicity, take the case of a single mean in the usual conditions of unknown population variance. In a two-sided size-α test of the null hypothesis *H*_0_: μ = μ_0_ against the alternative *H*_1_: μ ≠ μ_0_, the null is *not* rejected if it so happens that *t*_*n*−1,α/2_ < *T* < *t*_*n*−1,1−α/2_, where *T* = $\frac{\bar{X}-\mu_{0}}{s_{x}/\sqrt{n}}$, $\bar{X}$ is the sample mean, *s*_*x*_ is the sample standard deviation, *n* is the sample size, and *t*_ν_,_*q*_ is the *q*-th quantile of a *t* distribution on ν degrees of freedom. The reason is that the sampling distribution of the test statistic *T* ensures that

(1) $$\begin{array}{cc} \text{Prob}(t_{n-1,\alpha /2}<T<t_{n-1,1-\alpha /2})=1-\;\alpha & \;(1) \end{array}$$

if the null hypothesis is correct (i.e., if the true value of the population mean is μ_0_), rendering the desired size-α test. It is important to notice that *T* in Equation (1) is a random variable and, then, Equation (1) represents a statement about the probability that such a random variable lies within the stated limits.

One can thus wonder about the set of hypotheses (i.e., values of μ_0_) that the current data would not reject. Rather than repeatedly computing *T* for different values of μ_0_ and checking against the limits for rejection, one can manipulate Equation (1) algebraically to arrive at

(2) $$\begin{array}{cc} \text{Prob}(\bar{X}-\frac{s_{x}}{\sqrt{n}}t_{n-1,1-\alpha /2}<\;\mu_{0}<\bar{X}-\frac{s_{x}}{\sqrt{n}}t_{n-1,\alpha /2})=1-\alpha , & \;(2) \end{array}$$

which thus gives the well-known limits of the 100(1 − α) % CI for the mean.

It is important to steer away from two common misconstructions of Equation (2). First, μ_0_ (the hypothesized value) cannot be replaced with μ (the true mean) in Equation (2). The true value of the population mean was never involved in this derivation and it cannot appear in it by magic. Because the algebraic manipulation that rendered Equation (2) cannot alter the original meaning of Equation (1), this manipulation has only made explicit the range of values for μ_0_ that the current data will not reject. Second, Equation (2) is not a statement about the probability that μ_0_ (let alone μ) lies within the stated limits, because μ_0_ (let alone μ) is not a random variable in the first place. Then, the *literal* interpretation of Equation (2) (or the corresponding CI) as expressing the probability that the true mean lies between those limits is unjustifiable and incorrect: The true mean is a fixed value, not a random variable with some probability distribution. This holds also for analogous statements that can be made from other theoretical frameworks. From the perspective discussed thus far, a CI is only the range of values that one could have placed in a null hypothesis that the current data would not reject in the corresponding size-α test.

The foregoing discussion used a two-sided test and its dual CI but analogous considerations hold for one-sided tests, although CIs associated with one-sided tests illustrate more clearly the inadequacy of some common misinterpretations of CIs. Consider a right-tailed size-α test of *H*_0_: μ = μ_0_ against *H*_1_: μ > μ_0_. Now the null is *not* rejected if it so happens that *T*<*t*_*n*__−1, 1−α_, also because

(3) $$\begin{array}{cc} \text{Prob}(T<t_{n-1,1-\alpha })=1-\;\alpha & \;(3) \end{array}$$

if the null is correct. Algebraic manipulation of Equation (3) renders

(4) $$\begin{array}{cc} \text{Prob}(\mu_{0}>\bar{X}-\frac{s_{x}}{\sqrt{n}}t_{n-1,1-\alpha })=1-\alpha . & \;(4) \end{array}$$

Derivation of the CI associated with a left-tailed test is analogous and the resultant CI has infinite width in both cases. Again, although Equation (3) is unquestionably a statement about the probability that the random variable *T* falls below *t*_*n*−1, 1−α_, Equation (4) only describes the range of values for μ_0_ that one could place in a null hypothesis that the current data would not reject. A numerical example shows also that the common misinterpretation that a CI indicates the plausible range where the true parameter lies is unacceptable. Consider a size-0.05 test of *H*_0_: μ = 100 against *H*_1_: μ > 100 using a random sample of *n* = 50 observations for which $\bar{X}=101.71$ and *s*_*x*_ = 9.07. The data do not reject the null (*T* = 1.331; *p* = 0.095); on the other hand, from Equation (4), the 95% CI for the mean ranges from 99.56 to infinity. These two results are in apparent contradiction if one mistakes the CI as indicating a range of “plausible” values for the true mean: Given that the CI includes also the value 150, why was the null not rejected in the right-tailed test? The obvious answer is that the CI only indicates the range of hypotheses that the current data do not reject. Clearly, these data do not reject *H*_0_: μ = 150 against *H*_1_: μ > 150 because, for one thing, the sample mean is away from the null in a direction opposite to what the alternative states. This example also shows once again that not rejecting the null hypothesis does not mean that the null is true (or even reasonable).

CIs can be derived that are dual with significance tests for other parameters (variances, Bernoulli probabilities, correlations, etc.) or functions thereof (e.g., differences between means). All of them can always be interpreted as the range of parameter values that the current data would not have rejected. Whenever hypothesis testing is the primary research goal, this interpretation of CIs softens the apparent magical status of a point null hypothesis (e.g., that the difference between population means is exactly 0) and broadens the researcher's view by making explicit that there is a range of hypotheses that the data are compatible with (in the sense of not being rejected by the data).

### The CI as a claim (about the parameter) that is true with probability 1 − α

CIs constructed as discussed above have the property that 100(1 − α) % of them include the true value of the parameter (or the difference between parameters). This property stems from the sampling distributions also used to define non-rejection regions in significance tests. But we will never know whether the CI computed from the data on hand is one of those that include the value of the population parameter. Any particular CI, with its limits computed from the current data, is the observed outcome of a random experiment and it either includes the parameter or it does not: There is no such thing as a probability that *this particular CI* includes the parameter.

To see that probabilities are not attached to observed outcomes, consider the textbook example of a random experiment in which a ball is drawn from an urn in which 95% of the balls are red and the rest are white. The Bernoulli random variable is “color of the drawn ball,” with a probability distribution in which “red” has probability 0.95 and “white” has probability 0.05. Drawing a ball renders a particular realization that is invariably red or white, not red with some probability and white with the rest. Understanding the distinction between a random variable (with values to which probabilities of occurrence can be assigned) and its realizations (which are the specific values that happened to be observed and whose status is that of invariable facts) allows one to accept without hesitation that the probability of drawing a red ball is 0.95 and, simultaneously, that each individual ball in the urn is invariably red or white; no-one would claim that this ball just pulled out of the urn, which is unmistakably white, still has 0.95 probability of being red. In other words, the color of the ball that happened to be drawn on an individual occasion is not a random variable but a fact.

Conducting a study to estimate distributional parameters represents an analogous random experiment: One draws a sample from the population of samples that could have been drawn, and this sample “comes with” a realization of the, say, 95% CI for the parameter. The relevant Bernoulli random variable in the context of this discussion is “true parameter value enclosed by the CI,” which also has a discrete probability distribution in which “yes” has probability 0.95 and “no” has probability 0.05. But, unlike in the urn example, looking at the computed CI does not allow identifying whether the outcome was “yes” or “no” and, yet, it is definitely one or the other. In other words, the realization of the random experiment is not classifiable. Regarding the interpretation of CIs in these conditions, the crucial question is, then, what is 1 − α the probability of? Certainly, it is not the probability that the observed CI encloses the true parameter value because, like the ball drawn from the urn, this CI either encloses or does not enclose the parameter. And, at the same time, the random experiment was realized under conditions in which CIs that enclose the true parameter value occur with probability 1 − α.

The tricky part is embedding these notions in an exquisitely precise but succinct and non-convoluted statement that, ideally, does not lend itself to misinterpretations. It is nevertheless puzzling that the misinterpretation of statements on the probability with which random events may occur hits so hard the community of researchers in psychology when it comes to CIs. Consider a thoroughly analogous situation in a different context, namely, someone buys a single ticket for a 1000-number, single-prize raffle. Until the day of the draw, one can only state that the probability is 0.001 that this will be the winning number. On the day of the draw, the ticket either holds or does not hold the winning number, an invariable fact to which probability no longer applies. This transition requires that the draw takes place and, importantly, that its outcome is identifiable. Yet, before the draw, one lives in comfortable harmony with the idea that “the probability that I have the winning number is 0.001.” Computing a, say, 95% CI is essentially identical to buying a raffle ticket that has 0.95 probability of being a winner (i.e., enclosing the true parameter value), but with a crucial difference: The final draw is never made, as this will require that at some point we come to know the true parameter value. In the raffle, hardly anybody will mistake the statement “the probability that I have the winning number is 0.001” as meaning that the winning number pulled out in the final draw has a probability 0.001 of being the number that it actually is. Why psychologists find trouble dealing with the analogous statement “the probability is 0.95 that the 95% CI that I just computed encloses the true parameter” is anybody's guess, but the reason may be a failure to realize that a computed CI is like a ticket for a raffle in which the final draw is endlessly postponed. Or, perhaps more fittingly, like a scratch-off ticket that cannot be scratched to disclose whether the prize was won. The key issue is that computing a CI is not observing the outcome of the random variable of concern (i.e., enclosure of the true parameter), which is indeed unobservable. Then, the only specificity around the computation of CIs is that none of the individual realizations of the random experiment can ever be classified as to enclosure. But this does not change the characteristics of the Bernoulli random variable of concern, nor does it affect the probability distribution of its outcomes. These considerations lead to the statement that we were after.

Consider the population of, say, 95% CIs for the mean computed across all samples that could be drawn in the context of some study. For each sample, the CI ranges from A to B, with A and B varying across samples. The properties of CIs ensure that 95% of the members of the population of CIs enclose the true parameter value and, hence, the claim “the true mean lies between A and B” is true for 95% of the members, just as the claim “the ball is red” is true for 95% of the balls in the urn or the claim “this is the winning number” is true for 0.1% of the raffle tickets. Conducting a study amounts to drawing a single sample that provides the data for all computations, including the 95% CI for the mean. Then, the claim “the population mean lies between A and B” has been randomly drawn from a population of claims in which 95% of them are true and, hence, such claim has probability 1 − α of belonging in the subset of claims that are true. Thus, the statement “the 95% CI for the mean ranges between 0.1 and 0.4” means “the claim that the population mean lies between 0.1 and 0.4 has 0.95 probability of being true.”

In sum, the procedure used to compute CIs ensures that the categorical claim “Parameter θ lies between A and B” is true with probability 1 − α. The limits A and B will vary across samples from the same population and also with the size of each sample. When one such claim is made with A and B computed from the current data, that particular realization of the claim is certainly either true or false but its truth value will never be unraveled, as in the endlessly postponed raffle. The probability 1 − α applies to the truth of the claim that the parameter lies within the stated range.

The use of CIs for interval estimation raises another issue that needs to be discussed due to its bearings on Hoekstra et al.'s (2014) items. In principle, CIs could be derived to be dual with two-sided, right-tailed, or left-tailed tests. The procedure ensures that the resultant claims are true with probability 1 − α, but the stated ranges differ meaningfully. Consider the numerical example given in the preceding section, where a right-tailed 95% CI yields the claim “The true mean is greater than 99.56.” For the same data, a left-tailed 95% CI yields “The true mean is lower than 103.86” whereas a two-sided 95% CI yields “The true mean lies between 99.13 and 104.28.” Without sacrificing the probability that the claim is true (i.e., keeping 1 − α fixed), one might strive for the CI involving the narrowest range. When the parameter of concern is the mean (or the difference between means), the narrowest range occurs indeed for the *central* or *equal-tails* CI associated with a two-sided test, where the upper and lower limits are computed by splitting α evenly between the left and right tails (as in Equation 1 above). An infinite number of noncentral CIs can be defined by instead splitting α unevenly, of which the left-tailed CI and the right-tailed CI are the most extreme cases. Interestingly, central CIs are not the narrowest members of this infinite family when they are based on skewed sampling distributions, and this is the case when the parameters of concern are variances, Bernoulli probabilities, or correlations. In general, for any split of α into ε_1_ and ε_2_ such that ε_1_ ≥ 0, ε_2_ ≥ 0, and ε_1_ + ε_2_ = α, the 100(1 − α) % CI is

(5) $$\begin{array}{cc} \left[\bar{X}-\frac{s_{x}}{\sqrt{n}}t_{n-1,\;1-\varepsilon_{2}},\;\bar{X}-\frac{s_{x}}{\sqrt{n}}t_{n-1,\varepsilon_{1}}\right] & \;(5) \end{array}$$

for the mean,

(6) $$\begin{array}{cc} \left[\frac{ns_{x}^{2}}{\chi_{n-1,1-\varepsilon_{2}}^{2}},\;\frac{ns_{x}^{2}}{\chi_{n-1,\varepsilon_{1}}^{2}}\right] & \;(6) \end{array}$$

for the variance, where $\chi_{v,q}^{2}$ is the *q*-th quantile of a χ^2^ distribution on *v* degrees of freedom,

(7) $$\begin{array}{cc} \begin{array}{l} {}[\frac{2np+z_{1-\varepsilon_{2}}^{2}-z_{1-\varepsilon_{2}}\sqrt{4np(1-p)+z_{1-\varepsilon_{2}}^{2}}}{2(n+z_{1-\varepsilon_{2}}^{2})},\;\\ \;\frac{2np+z_{\varepsilon_{1}}^{2}-z_{\varepsilon_{1}}\sqrt{4np(1-p)+z_{\varepsilon_{1}}^{2}}}{2(n+z_{\varepsilon_{1}}^{2})}] \end{array} & \;(7) \end{array}$$

for the Bernoulli probability, where *p* is the sample proportion and *z*_*q*_ is the *q*-th quantile of the unit-normal distribution,^1^ and

(8) $$\begin{array}{cc} \left[\frac{1-\frac{1-r_{xy}}{1+r_{xy}}\exp\;\left[\frac{2z_{1-\varepsilon_{2}}}{\sqrt{n-3}}\right]}{1+\frac{1-r_{xy}}{1+r_{xy}}\exp\;\left[\frac{2z_{1-\varepsilon_{2}}}{\sqrt{n-3}}\right]},\;\frac{1-\frac{1-r_{xy}}{1+r_{xy}}\;\exp\;\left[\frac{2z_{\varepsilon_{1}}}{\sqrt{n-3}}\right]}{1+\frac{1-r_{xy}}{1+r_{xy}}\exp\;\left[\frac{2z_{\varepsilon_{1}}}{\sqrt{n-3}}\right]}\right] & \;(8) \end{array}$$

for the correlation, where *r*_*xy*_ is the sample correlation.

**Figure 1:**
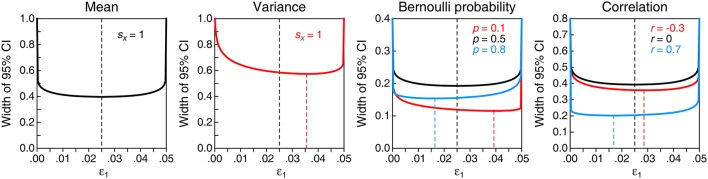
**Width of the 95% CI for several parameters (panels) as a function of how α (0.05 in this illustration) is split into ε_**1**_ and ε_**2**_, with ε_**1**_ + ε_**2**_ = α**. In each panel, the horizontal axis indicates the value of ε_1_ and the dashed vertical line marks the even split that renders the central CI. The curve(s) in each panel plot(s) the width of the CI defined as the difference between the upper and lower limits in Equations (5)–(8), with values for required sample statistics indicated in the insets in each panel and assuming *n* = 100 in all cases. The precise split at which the CI is shortest is indicated by a dashed vertical line from the curve (at its minimum) to the horizontal axis. CIs represented with black curves are shortest at the even split.

In the above expressions, ε_1_ = ε_2_ = α/2 renders the central CI whereas ε_1_ = 0 (hence, ε_2_ = α) renders the right-tailed CI and ε_1_ = α (hence, ε_2_ = 0) renders the left-tailed CI. As seen in Figure 1, the central CI is shortest for the mean, for the Bernoulli probability when the sample proportion is 0.5, and for the correlation when the sample correlation is 0; in other cases, there is always some noncentral CI that is shorter than the central CI, although which one it is varies with sample size and with the values of the sample statistics involved in the computation. Although short CIs are comforting, it should be noted that they are not more accurate: The probability that the resultant claim about the parameter is true is always 1 − α, regardless of the width (or location) of the CI.

### Inferring misinterpretation from endorsement of items in the questionnaire

Consideration of the two correct interpretations of a CI allows an assessment of the certainty with which misinterpretation of CIs can be inferred from endorsement of the items in the questionnaire of Hoekstra et al. (2014). Inferring states of knowledge from item responses requires items worded unambiguously and whose content relates exclusively to the piece of knowledge being assessed and not to something else. We argued in the Introduction that items 3 and 6 in the questionnaire do not satisfy at least one of these requirements and we will not discuss them further here, but recall that the statement in item 3 is correct (on grounds unrelated to CIs) whereas the statement in item 6 is incorrect on grounds also unrelated to CIs. In these conditions, inferring correct or incorrect interpretation of a CI from responses to these two items takes a leap of faith.

Besides the two conditions just stated, items must be administered with allowance for omissions, because uninformed responses are unrelated to the knowledge being assessed. In our discussion of the four remaining items, we will assume that responses are informed (or misinformed, for that matter) when we refer to the states that can be inferred from endorsement or non-endorsement.

We will leave the first two items for last. Items 4 and 5 (namely, “there is a 95% probability that the true mean lies between 0.1 and 0.4” and “we can be 95% confident that the true mean lies between 0.1 and 0.4”) only differ in a reference to probability or confidence. Hoekstra et al. (2014) assumed that these items would be endorsed by those who think that probability or confidence relates to the stated range of values for the parameter, whereas those who understand that this is not the case would not endorse the items. However, there is nothing in the wording of these items that prevents knowledgeable respondents from interpreting that probability (or confidence) refers instead to the truth of the claim being made (as in, e.g., “there is a 0.1 probability that the winning number is among the 100 raffle tickets in my hands”). They would thus demonstrate their correct interpretation of CIs by endorsing both items. In other words, the imprecise wording of items 4 and 5 carries two alternative meanings, only one of which is incorrect as an interpretation of CIs. But which meaning respondents reacted to is undecipherable from their response.

Item 1 (namely, “the probability that the true mean is greater than 0 is at least 95%”) is equally ambiguous, raising the same issues as items 4 and 5. A knowledgeable respondent (i.e., one who interprets this item as “the claim ‘*the true mean is greater than 0*’ is true with probability 0.95 or higher”) will realize that the information provided is insufficient to respond. From Equation (4) above, item 1 is true at the limiting probability if $\bar{X}-\frac{s_{x}}{\sqrt{n}}$*t*_*n*−1, 0.95_ = 0. In search for the answer, one may surmise that, as usual, Prof. Bumbledorf was reporting the *central* 95% CI in which ε_1_ = ε_2_ = α/2 so that $\bar{X}=0.25$. But the remaining ingredients are nowhere to be found, although it is virtually impossible that the numbers will match up. Then, to a knowledgeable respondent, item 1 is likely (subjectively) to be false, but not for the reason that Hoekstra et al. (2014) intended to embed in it.

Item 2 (i.e., “the probability that the true mean equals 0 is smaller than 5%”) is equally ambiguous and poses a similar challenge to knowledgeable respondents who take it as meaning “the claim ‘*the true mean equals 0*’ is true with probability less than 0.05.” The solution is simpler in this case. By mentioning a single value instead of a range, the claim relates to the (central or noncentral) zero-width CI associated with α = 1 (i.e., ε_1_ + ε_2_ = α = 1 makes *t*_*n*−1, 1−_ε__2__ = *t*_*n*−1,_ε__1__ in Equation (5) and, thus, the CI has zero width). Hence, the claim “the population mean equals 0” is true with probability 1 − α = 0 and, since 0 < 0.05, the knowledgeable respondent will endorse this item.

In sum, inferring misinterpretation from endorsement of the nominally incorrect statements in the questionnaire of Hoekstra et al. (2014) is difficult to justify. Some items can be identified as incorrect (and, hence, not endorsed) irrespective of their reference to CIs, whereas others are correct (and, hence, endorsable) when the ambiguity of their wording is resolved in the appropriate manner, something that rests entirely in the hands of the respondent without leaving any observable traces. Hoekstra et al.'s conclusion that CIs are robustly misinterpreted is, then, unwarranted.

## A closer look at the data of Hoekstra et al. (2014)

Hoekstra et al. (2014) administered the 6-item questionnaire we just discussed to 442 first-year psychology students, 34 master students, and 120 researchers, although responses from two of the latter were discarded and results reported for the remaining 118 researchers. (Interestingly, these individuals were excluded because one of them had left an item unanswered and the other had indicated that one of the items was both true and false.) All items were regarded as incorrect and the main outcome variable was the number of items endorsed (NE). The average NE was similar in all groups (3.51, 3.24, and 3.45, respectively for first-year students, master students, and researchers) despite their presumed differences in knowledgeability.

**Figure 2:**
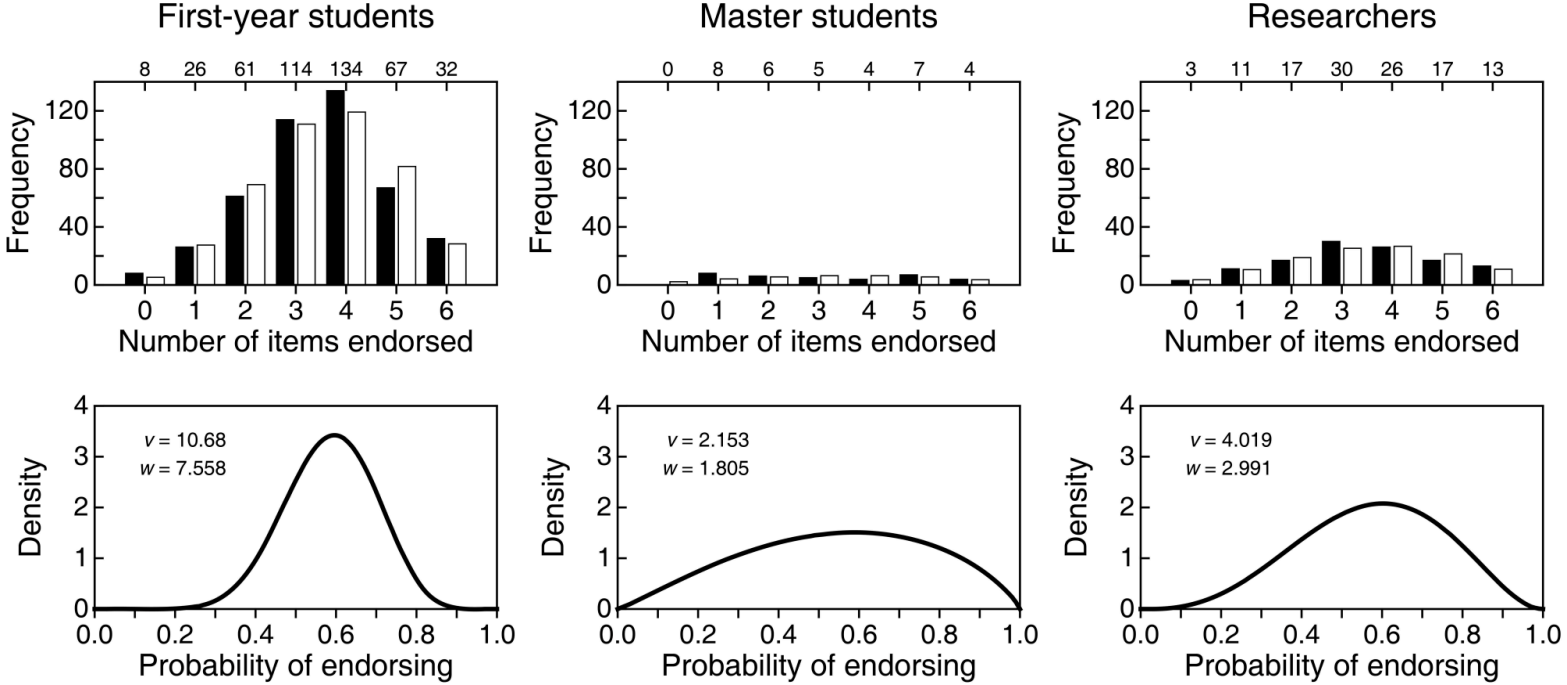
**(Top row)** Empirical distribution (solid bars) of number of items endorsed (NE) in each of three groups and expected distribution (open bars) in each group according to a beta-binomial model. Numerals along the top of each frame indicate the empirical count of respondents at each NE. **(Bottom row)** Estimated beta distribution for the probability *p* of endorsing in each group, with estimated parameters printed in the insets.

Even if all items were incorrect, a mere comparison of average NE overlooks some differences that can be appreciated in the raw distributions of NE (solid bars in the top row of Figure 2). While the distribution for first-year students is peaked and narrow, distributions for master students and researchers are broader and less peaked, particularly for master students. This shows once more that routine comparison of means is not always the best way to compare groups. To assess these differences, we conducted a size-0.05 omnibus test of homogeneity of distributions, which came out significant ($X_{12}^{2}$ = 24.81; *p* = 0.016). A size-0.05 familywise test with correction for multiple testing in the analysis of residuals (García-Pérez et al., 2015) also rejected homogeneity due to larger-than-expected counts at extreme NE for master students. Although significance tests do not reveal truth, these results indicate that the noticeable difference in the distribution of NE in Figure 2 for master students relative to the two other groups is larger than one would generally expect from sampling error if the population distributions were identical.

Because the empirical distributions are overdispersed for a binomial variable, we fitted a beta-binomial model in which the probability *p* of endorsing has a beta distribution with parameters *v* and *w* (Wilcox, 1981; see also Forbes et al., 2011, p. 61). We obtained maximum-likelihood estimates of these parameters in each group and the estimated beta distributions for *p* are plotted in the bottom row of Figure 2. Open bars in the top row of Figure 2 depict the expected distribution under the fitted beta-binomial model. The fit is good by eye and, in size-0.05 tests, the *G*^2^ goodness-of-fit statistic with four degrees of freedom did not reject the model in any group (first-year students: *G*^2^ = 7.40, *p* = 0.116; master students: *G*^2^ = 8.99, *p* = 0.061; researchers: *G*^2^ = 2.55, *p* = 0.635).

Whether via empirical distributions of NE (solid bars in the top row of Figure 2) or via the estimated distributions of *p* (bottom row of Figure 2), one cannot help wondering why master students would be so different. In principle, statistical education should have made them knowledgeable in comparison to first-year students who have not yet received such education. Then, if statistical education is effective, one would expect the distribution of NE to be shifted downward for master students compared to first-year students: The latter will only display the willingness to endorse statements by incognizant respondents, whereas master students (and researchers) should have been able to identify the statements in the questionnaire as incorrect. Compared to the beta distribution for first-year students (left panel in the bottom row of Figure 2), the probability mass is indeed larger at low endorsement rates for master students and researchers, but it is at the same time also larger at high endorsement rates. Ironically, knowledgeability may indeed be responsible for this puzzling outcome, given that some of the (only nominally) incorrect statements could have been identified as correct by knowledgeable respondents and, hence, endorsed.

Unfortunately, Hoekstra et al.'s (2014) questionnaire does not permit assessing knowledgeability, that is, whether the endorsement patterns of master students and researchers reflect incognizance, correct interpretation, or misinterpretation. What is nevertheless clear is that first-year students' responses do not reflect misinterpretation but only their willingness to endorse when forced to classify items as true or false under complete ignorance.

## Our replication

We replicated Hoekstra et al.'s (2014) study with extensions that permit telling misinterpretation from incognizance and identifying correct interpretations of CIs. This was achieved by altering the response format and by including items that unequivocally express correct statements. Our study included samples of first-year and master students only. Data from first-year students were not expected to reveal anything but their incognizance. Yet, and precisely for this reason, their data served the more important purpose of checking out whether correct and nominally incorrect statements differ in attractiveness, something that would result in method bias. To parallel the analyses of Hoekstra et al., the six items in the original questionnaire were grouped together as nominally incorrect items, but recall that some of them are actually correct whereas others are correct or incorrect according to how their ambiguity is resolved.

### Participants

The sample of first-year students consisted of 313 individuals enrolled in a bachelor program in psychology at Universidad Complutense de Madrid (UCM). At the time of our study, they were taking an introductory statistics course that did not include any inferential statistics. The sample of master students consisted of 158 individuals enrolled in a master program in psychology at UCM. All master students had taken a course on inferential statistics during their bachelor studies and they had also had numerous opportunities to come across CIs as presented in the literature within their specialty choice.

### Materials and procedure

This research followed the APA ethics code and the protocol was institutionally approved. We used a Spanish translation of the questionnaire of Hoekstra et al. (2014), which was supplemented with two additional items expressing correct interpretations of CIs (see the Appendix in Supplementary Material). Our questionnaire was otherwise administered with identical instructions. To facilitate cross-references, the first six items were ordered as in the original questionnaire and the two additional items stated (in Spanish) the following:

7. The claim “The true mean lies between 0.1 and 0.4” is true with probability 0.95.
8. The data are compatible with the notion that the true mean lies between 0.1 and 0.4.

These items express the two correct interpretations discussed earlier. The last item is admittedly vague insofar as the referents for “compatible” and “notion” are unclear, and also because it does not link up with the confidence level. Nevertheless, there is nothing strictly incorrect in this vague wording. With only two correct statements, our 8-item questionnaire is still imbalanced but we decided against including other correct statements that do not truly assess interpretation of CIs.

The questionnaire was administered during lecture sessions in several sections of first-year and master students. Participation was voluntary and anonymous. Instructions encouraged answering all items by stating that sheets with omissions would be useless to us. Once master students in a section had completed the questionnaire, they were immediately asked to place a mark next to the items that they would rather have omitted or where they would have ticked an “I don't know” response box had it been provided. The response sheet did not include anything suggestive of a second pass and students had not been forewarned of it. This is why second-pass data are unavailable for 14 of the 158 master students, as they had left the room before the second pass started. We did not ask students to indicate the reasons why they marked items on the second pass, as we only wanted to identify uninformed responses that only reflect guesses motivated by the instructions to answer all items. This second pass was meant to collect more useful information than Hoekstra et al.'s (2014) measure of self-reported expertise, which surely reflects the Dunning-Kruger effect (Kruger and Dunning, 1999; Williams et al., 2013). The second pass was omitted with first-year students because it was clear that hardly any of them could have given informed responses.

## Results

Data for the subsets of (nominally) incorrect and correct items will be reported separately under the same method of analysis used above for the data of Hoekstra et al. (2014). The results of a joint analysis of the two subsets of items as well as the results for informed responses identified in the second pass with master students will be described subsequently. Data are available as Supplementary Material.

**Figure 3:**
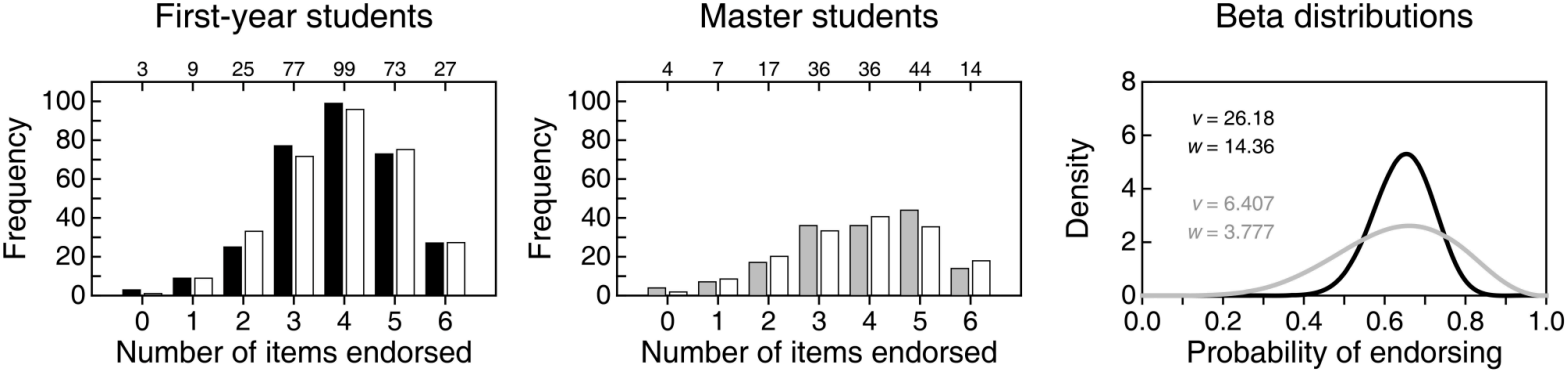
**Empirical (solid or gray bars) and expected (open bars) distributions of number of items endorsed (NE) by 313 first-year students (left panel) and 158 master students (center panel) on the six items that our questionnaire shared with that of Hoekstra et al. (2014)**. Numerals along the top of each frame indicate the empirical count of respondents at each NE. The right panel shows the estimated beta distributions for the probability of endorsing by first-year students (black curve) and by master students (gray curve), with parameters printed in the inset. Size-0.05 goodness-of-fit tests with four degrees of freedom did not reject the beta-binomial model for any of the groups (first-year students: *G*^2^ = 4.95, *p* = 0.292; master students: *G*^2^ = 6.05, *p* = 0.196).

Figure 3 shows the distributions of NE among the first six items. The distribution for first-year students (solid bars in the left panel) is similar to that reported by Hoekstra et al.'s (2014; compare with the left panel in the top row of Figure 2) surely because, anywhere, first-year students who have never heard of CIs can only respond on the basis of intuition and common sense. In other words, these responses only reflect endorsement in conditions in which admitting to nescience and omitting is not allowed. As it turns out, the probability that first-year students endorse items meanders around 0.65, as indicated by the estimated beta distribution in the right panel of Figure 3 (black curve). For Hoekstra et al.'s data, the estimated beta distribution had a similar location but was slightly broader (see the bottom panel in the left column of Figure 2). Why first-year students are more prone to endorsing than to not endorsing items in either study is unclear but, obviously, these results do not reflect misinterpretation of CIs but plain (and understandable) incognizance.

The distribution of NE for master students (gray bars in the center panel of Figure 3) is broader and more negatively skewed. Accordingly, the estimated beta distribution of probability of endorsing (gray curve in the right panel of Figure 3) is broader relative to that of first-year students (black curve in the same panel). This difference between first-year and master students as to NE is analogous to that in the samples of Hoekstra et al. (2014), although their data rendered a more uniform distribution for master students (compare with Figure 2). The fact that presumably knowledgeable master students display in both studies similar probability of endorsing compared to first-year students might be taken as a sign that something in the teaching of statistics (or of CIs in particular) produces a diversification whereby, in comparison to the endorsing behavior of nescient first-year students, some master students indeed endorse fewer incorrect statements but others endorse more. This is, of course, on the (wrong) assumption that these items express incorrect interpretations. A look at endorsing patterns on items that express correct interpretations should be more informative.

**Figure 4:**
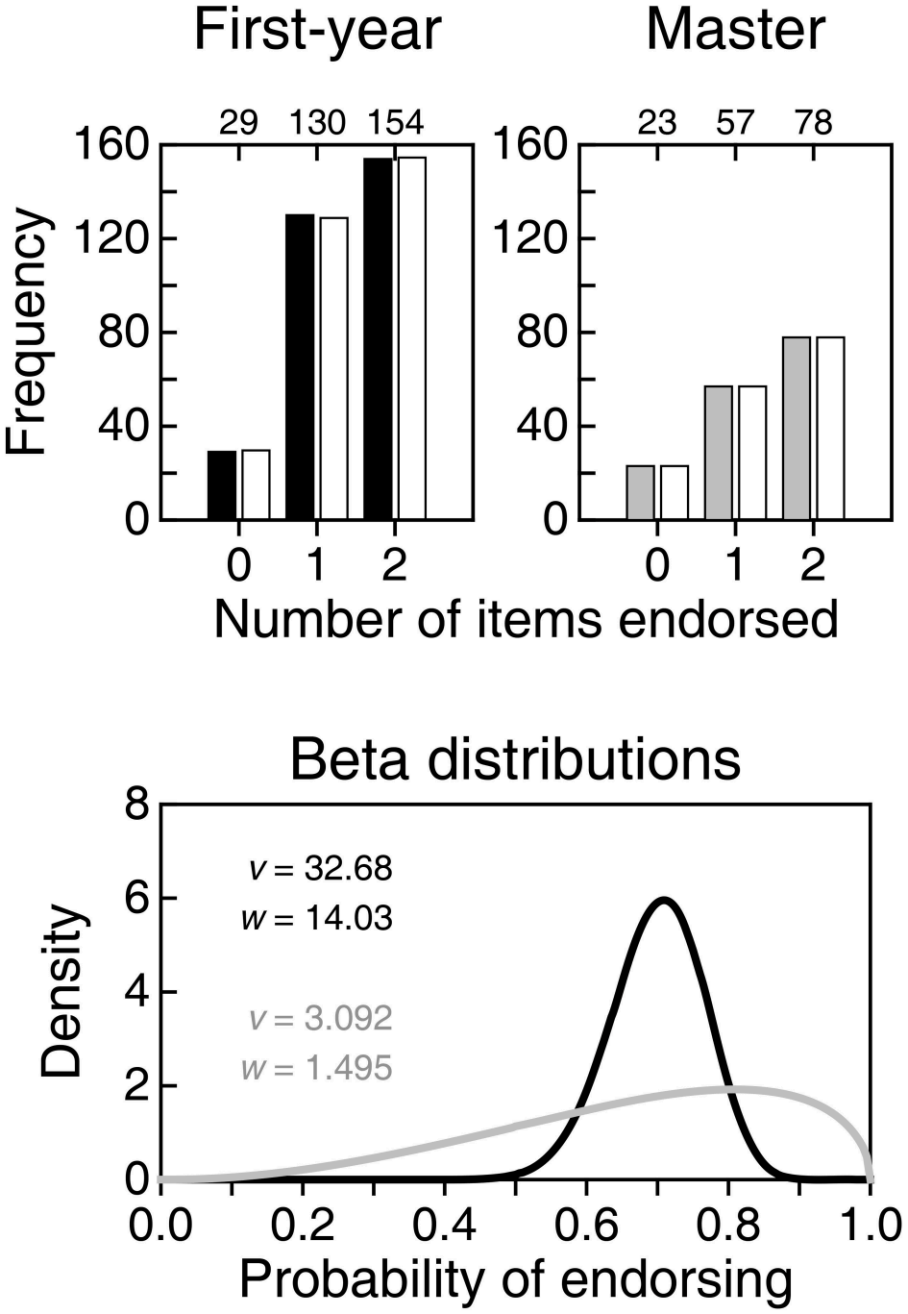
**(Top)** Empirical (solid or gray bars) and expected (open bars) distributions of number of items endorsed (NE) by 313 first-year students **(left panel)** and 158 master students **(right panel)** on the two items that expressed correct interpretations of a CI. Numerals along the top of each frame indicate the empirical count of respondents at each NE. **(Bottom)** Estimated beta distribution for the probability of endorsing by first-year students (black curve) and by master students (gray curve), with parameters printed in the inset. With three categories and two parameters to estimate, there are no degrees of freedom available to test the fit of the beta-binomial model.

Figure 4 shows an analogous analysis of the two correct items. In contrast to the preceding set of items, understanding CIs would result here in large endorsement rates. The distribution of NE for first-year students (top-left panel) renders an estimated distribution for the probability of endorsing (black curve in the bottom panel) that is nearly identical to that for the six other items (compare with the black curve in the right panel of Figure 3). This similarity is expected and unsurprising, since statements that reflect correct vs. incorrect interpretations of a CI should be indistinguishable to respondents who do not know what a CI is. The similarity of the beta distributions of the probability of endorsing incorrect vs. correct items by first-year students strengthens the conviction that their data reflect only the “willingness to endorse” of unknowing respondents. But, more importantly, this confirms that there are no spurious differences between our correct items and the (nominally) incorrect original items that might make one set more attractive than the other to unknowing respondents. Hence, endorsing correct items more often than (nominally) incorrect items is an indication of correct interpretation of CIs.

The distribution of NE for master students on correct items is shown in the top-right panel of Figure 4 and renders an estimated beta distribution for the probability of endorsing (gray curve in the bottom panel of Figure 4) that shows the signs of correct interpretation of CIs: It differs markedly from the beta distribution for incorrect items (compare with the gray curve in the right panel of Figure 3), peaking at a higher probability of endorsing and with most of its mass in the upper range. These differences indicate that master students are not responding according to an undifferentiated willingness to endorse, as first-year students do. In fact, master students seem more prone to endorsing correct statements than to endorsing (nominally) incorrect statements, and suspicions mount that their endorsement of the latter may reveal that they simply identified some of the nominally incorrect items as actually correct. This result deserves further scrutiny by consideration of the information gathered in the second pass of the questionnaire, but we will first describe a joint analysis of the endorsement of correct and incorrect statements in each group of students.

**Figure 5:**
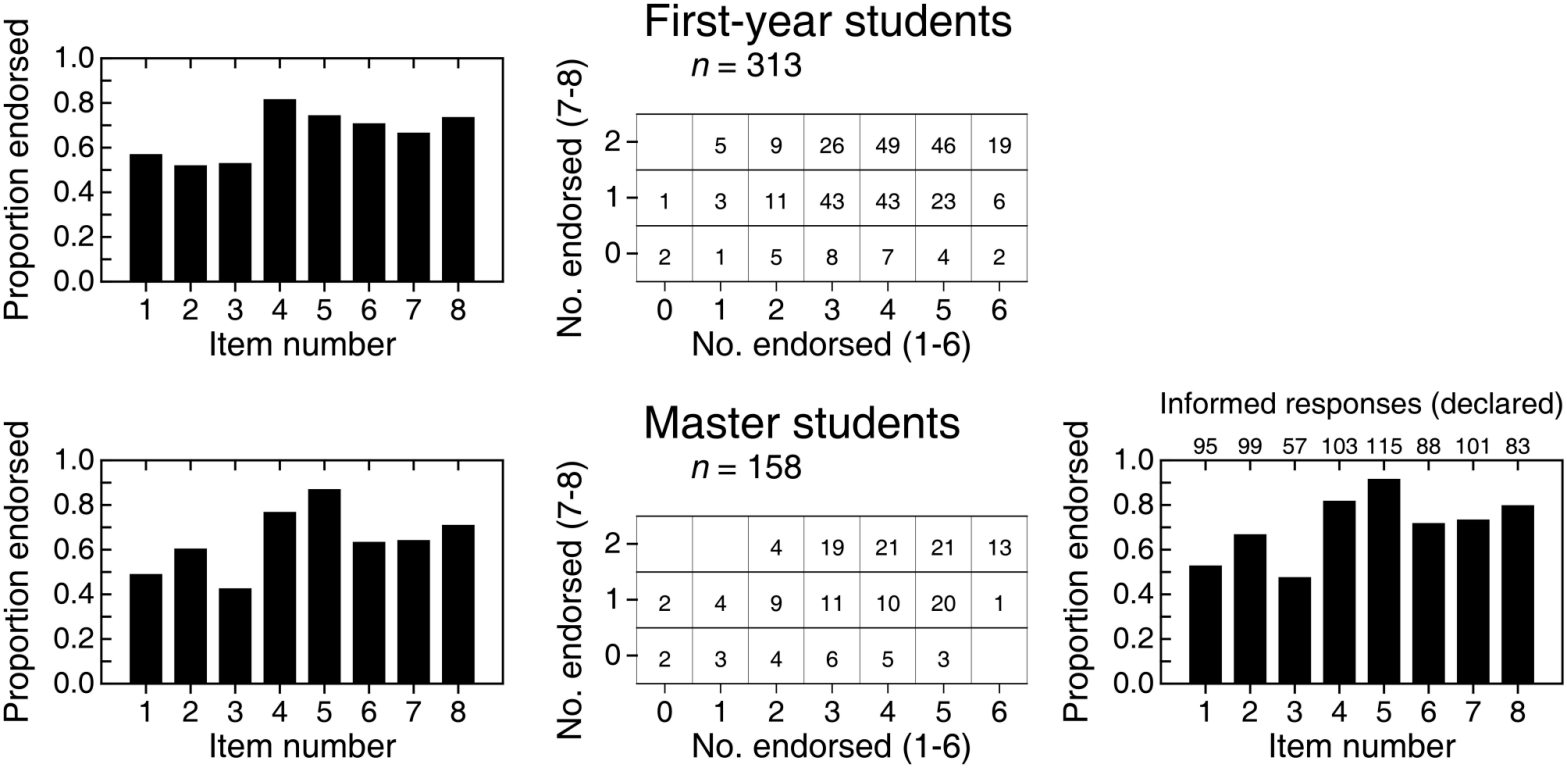
**Overall results for first-year students (top row) and master students (bottom row)**. **Left column**: Proportion of students endorsing each of the eight items on the questionnaire. **Center column**: Tabulated scatter plot of the number of correct items (items 7 and 8) endorsed against the number of incorrect items (items 1–6) endorsed. **Right column** (for master students only): Proportion of students endorsing each item after removing responses that had been marked during the second pass as uninformed. Numerals along the top of the frame indicate the raw numbers of responses on which these proportions are based.

The left column of Figure 5 shows endorsement proportions for each item individually. The correct items 7 and 8 are not conspicuously endorsed more often than the (nominally) incorrect items 1–6 in any of the groups. Yet, tabulated scatter plots of NE for correct items against NE for nominally incorrect items (center column in Figure 5) show that the top row (i.e., endorsing the two correct items) is relatively more populated than the second row (i.e., endorsing only one of the correct items) for master students. Although this difference indicates that master students depart from the pattern displayed by unknowing respondents, master students' data are still contaminated by uninformed responses that they had been forced to give. The second pass aimed at gathering information to remove uninformed responses (if any) for a proper assessment of misinformation. It is worth pointing out first that 18 (12.5%) of the 144 master students who responded on the second pass declared that they would rather not have responded true/false to any of the eight items whereas only 15 (10.4%) declared that they had given informed responses to all items. A meaningful amount of (self-declared) nescience thus exists among master students.

The right panel in the bottom row of Figure 5 shows proportion endorsed using only informed responses. Note that the number of informed responses for each item (numerals along the top of the frame) was remarkably low compared to 158 responses per item used in the leftmost panel, although those counts should be compared to 144 (i.e., the number of students who responded on the second pass). It is remarkable that item 3 was tagged in the second pass by 87 respondents (60.4%), which is not surprising in retrospect: As discussed earlier, item 3 is correct for reasons unrelated to CIs and, hence, a knowledgeable respondent is put to face the dilemma between endorsing a correct item and not endorsing it because its correctness is not logically linked to CIs. At the other end, item 5 (another nominally incorrect item whose ambiguous wording permits an interpretation that is correct) was tagged by only 29 respondents (20.1%) in the second pass. Interestingly, removal of uninformed responses results in endorsement rates that are uniformly higher (compare with the left panel in the bottom row of Figure 5), again with no noticeable difference between the nominally incorrect items 1–6 and the correct items 7 and 8.

**Figure 6:**
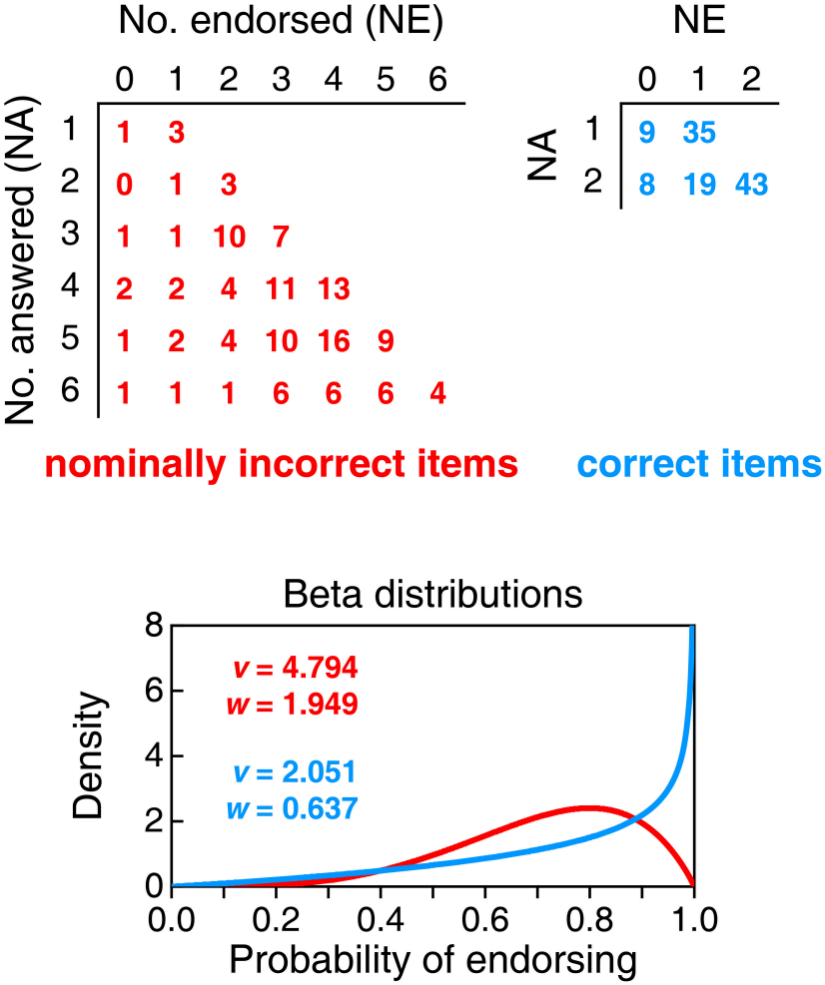
**Analysis of informed responses given by master students**. **Top:** Tabulated distributions of the number of items endorsed (NE) for each subsample defined by the number of items answered (NA). Tables are separately provided for nominally incorrect items **(left panel)** and correct items **(right panel)**. **(Bottom)**: Estimated beta distributions of the probability of endorsing nominally incorrect items (red curve) and correct items (blue curve), with parameters printed in the inset.

Figure 6 shows the results of an analysis of informed responses that leads to estimates of the beta distributions of probability of endorsing. Because this analysis requires computing NE anew for each respondent on each type of item once uninformed responses are treated as omissions, the sample splits into different subsamples according to how many items each master student actually answered (NA; between 1 and 6 for nominally incorrect items and between 1 and 2 for correct items, as students declaring that none of their responses were informed do not provide data for this analysis). The top part of Figure 6 tabulates the distribution of NE in each of the subsamples defined on NA (rows) for nominally incorrect items (left panel) and correct items (right panel). The applicable beta-binomial model varies with NA, but its underlying beta distribution of probability of endorsing should be unique for all NA for each item type. We thus sought maximum-likelihood estimates of the parameters of the beta distribution jointly across all applicable NA, with the results shown in the bottom panel of Figure 6. Size-0.05 goodness-of-fit tests did not reject the resultant beta-binomial model for any of the two groups of items (nominally incorrect items: *G*^2^ = 15.07, *p* = 0.718 with 19 degrees of freedom; correct items: *G*^2^ = 0.37, *p* = 0.545 with 1 degree of freedom).

The beta distribution for correct items (blue curve in the bottom panel of Figure 6) indicates proper interpretation of CIs in comparison with what this distribution was when uninformed responses were also included (compare with the gray curve in the bottom panel of Figure 4), but the distribution for nominally incorrect items (red curve in the bottom panel of Figure 6) also shifts upward in comparison to what it was with uninformed responses (compare with the gray curve in the bottom panel of Figure 3). This may seem to reveal confusion (i.e., endorsing correct and incorrect interpretations alike), but it is perhaps more fitting to see it as yet another sign that knowledgeable respondents disambiguate nominally incorrect items into correct statements that they endorse.

## Discussion

Our study provided data opposing Hoekstra et al.'s (2014) conclusion of a robust misinterpretation of CIs. First-year students are incognizant of CIs and their analogous endorsement distributions for incorrect and correct items indicate that their responses do not reflect misinterpretation but mere willingness to endorse, a response bias that cannot be identified when items are of the same type (all true or all false) and responding to all items is mandatory. In contrast, master students showed different endorsement patterns for correct and nominally incorrect items, ruling out a prevalent role of response bias even in the presence of forced responses. Although master students endorsed nominally incorrect items more often than might be expected, the beta distribution of their probability of endorsing correct items (gray curve in the bottom panel of Figure 4) has its mass shifted upward with respect to the corresponding distribution for nominally incorrect items (gray curve in the right panel of Figure 3). Yet, uninformed responses given in compliance with the instructions to answer all items necessarily contaminated these results; when uninformed responses were removed, the evidence of endorsement of correct interpretations of CIs pops out more clearly (blue curve in the bottom panel of Figure 6), although it coexists with evidence of endorsement of nominally incorrect interpretations (red curve in the same panel). The conclusion that master students endorse incorrect interpretations of CIs is unwarranted because the ambiguity of some of the nominally incorrect statements may have been resolved in a way that turns them into correct statements.

Questionnaires are not loyal and benevolent. They also do not necessarily gather data that can be unequivocally interpreted. Questionnaires with true/false items of only one type (whether all true or all false) do not permit assessing knowledge and misinformation independently: One of them is simply inferred by negation of the other. Furthermore, when administered with instructions to answer all items, uninformed responses interpreted at face value distort the resultant picture. Results reported in our Figure 4 (for forced responses) and our Figure 6 (for informed responses) reveal that Hoekstra et al. (2014) would surely have arrived at the opposite conclusion if they had included only correct statements among their items: relatively high endorsement rates, presumably indicative of correct interpretation of CIs even by unknowing first-year students.

Nevertheless, it is far from clear that passive endorsement of incorrect statements about a CI in a questionnaire reflects active misinterpretation or, analogously, that passive endorsement of correct statements reflects proper interpretation, even when omissions are allowed. We mentioned in the Introduction that scholars' interpretation of CIs manifests more reliably in their active descriptions to the effect or by analysis of reporting practices. Collecting such material is painstakingly slow, and interpreting verbiage to decipher what each respondent really meant is not without difficulties, but some attempts in that direction have been made (e.g., Cumming et al., 2004; Belia et al., 2005). In any case, inferring misinterpretation from such descriptions is questionable even in what seems to be the clearest of cases, illustrated next.

Recall that the average NE reported by Hoekstra et al. (2014) for first-year students, master students, and researchers was 3.51, 3.24, and 3.45, respectively. Miller and Ulrich (2016, p. 124) expressed this result as “students and researchers alike endorsed more than half of these statements as true.” This assertion only carries the incorrect meaning that each student and each researcher endorsed four or more statements. If such were the case, each mean would have exceeded 4.0, but notice also in the top row of Figure 2 that large numbers of respondents endorsed three or fewer statements. If we could take that producing an incorrect statement reflects misinterpretation, should not we conclude that Miller and Ulrich misinterpret the mean? Furthermore, given that their assertion appears to have gone unquestioned during the review process, should not we conclude also that such “tacit endorsement” reveals that the mean is robustly misinterpreted by all the reviewers of that paper and by all the colleagues who had reportedly read the manuscript outside the review process? Too long a shot. Most likely, this is just an instance of an unfortunate (active) wording on the part of Miller and Ulrich that readers and reviewers (passively) endorsed, resulting in an error that now graces the pages of a reputable journal. Since we understand this as an excusable mistake, on what basis can we positively infer misinterpretation in other cases? As this example shows, misinterpretation cannot be unequivocally inferred given the vagueness and ambiguities inherent to the natural language in which statements about CIs are expressed, and given also the habit of using for that purpose ready-made expressions that are admittedly unfortunate.

## Conclusion

Our replication of Hoekstra et al.'s (2014) study involved an extension with two correct items added to the questionnaire. This allowed us to identify that first-year students do not misinterpret CIs, not only because they had never heard of CIs but, more importantly, because their forced responses reflect the same willingness to endorse correct and nominally incorrect statements. This result with first-year students strengthens the confidence that different patterns of endorsement of correct and nominally incorrect items on an uncalibrated questionnaire inform of interpretations of CIs. Master students were more prone to endorsing correct than nominally incorrect statements under the forced-response format, and removal of uninformed responses resulted in a larger imbalance in favor of correct statements. The second pass of the questionnaire also revealed that incognizance of CIs (not to be confused with misinformation about them) is relatively prevalent among master students, as revealed by the fact that 12.5% of them marked all items on the second pass. Master students' informed responses still displayed non-negligible endorsement of nominally incorrect statements, but this may be a spurious outcome: Three of the nominally incorrect statements are sufficiently ambiguous to permit knowledgeable respondents to interpret them in a manner that endorsement reflects instead correct interpretation of CIs.

Unlike first-year students' responding essentially at random or according to how the wording of each statement sounds to them, master students' informed responses convey the effect of the statistical education that they had received. Our results show that such education was relatively efficacious in that they were more prone to endorsing correct than incorrect interpretations of a CI, and also on consideration that they may have endorsed nominally incorrect statements for a reason that indeed reveals proper interpretation. Our results also indicate a relative prevalence of incognizance of CIs on the part of master students. Identification of these three different states (incognizance, correct information, and misinformation) and quantification of their prevalence are essential for an assessment of the efficacy of statistical education and for the implementation of corrective measures (e.g., a revision of teaching methods to place more emphasis on the correct interpretation of CIs and the proper ways of wording it). The use of unbiased questionnaires is essential for this purpose, as is an adequate analysis of the data gathered with them.

Our results for a sample of students from a single institution show that misinterpretation of CIs is not as prevalent and widespread as Hoekstra et al. (2014) purported it to be, but this surely varies across institutions according to how statistics is taught in them. In the current context of statistical reform, giving today's students (i.e., tomorrow's researchers) proper education on CIs will surely be more efficacious than implementing remedial measures later. Training along the lines of our initial discussion about where CIs come from, how they are interpreted, and how such interpretations can be expressed in statements that unambiguously carry the proper meaning should be easy to implement in inferential statistics courses.

## Author contributions

All authors listed, have made substantial, direct and intellectual contribution to the work, and approved it for publication.

### Conflict of interest statement

The authors declare that the research was conducted in the absence of any commercial or financial relationships that could be construed as a potential conflict of interest.

## Footnotes

^1^This CI can be defined in other ways, but this is appropriate under binomial sampling (see García-Pérez, 2005).
