# Supplementary material for: The Interpretation of Scholars' Interpretations of Confidence Intervals: Criticism, Replication, and Extension of Hoekstra et al. (2014)
Source: Front Psychol. 2016 Jul 8;7:1042. doi: 10.3389/fpsyg.2016.01042 (PMC4937803; doi:10.3389/fpsyg.2016.01042)
Supplement: Supplementary file 2 [file DataSheet2.docx]

**Appendix**

This appendix lists (in English) the eight items on our questionnaire. The first six come directly from Hoekstra et al. (2014; see their Appendix 2); the last two are the additional items included in our extension.

1. The probability that the true mean is greater than 0 is at least 0.95.

2. The probability that the true mean equals 0 is smaller than 0.05.

3. The “null hypothesis” that the true mean equals 0 is likely to be incorrect.

4. There is a 0.95 probability that the true mean lies between 0.1 and 0.4.

5. We can be 95% confident that the true mean lies between 0.1 and 0.4.

6. If we were to repeat the experiment over and over, then 95% of the time the true mean falls between 0.1 and 0.4.

7. The claim “The true mean lies between 0.1 and 0.4” is true with probability 0.95.

8. The data are compatible with the notion that the true mean lies between 0.1 and 0.4.
